# Tomosyn associates with secretory vesicles in neurons through its N- and C-terminal domains

**DOI:** 10.1371/journal.pone.0180912

**Published:** 2017-07-26

**Authors:** Cornelia J. Geerts, Roberta Mancini, Ning Chen, Frank T. W. Koopmans, Ka Wan Li, August B. Smit, Jan R. T. van Weering, Matthijs Verhage, Alexander J. A. Groffen

**Affiliations:** 1 Department of Functional Genomics, Centre for Neurogenomics and Cognitive Research, Neuroscience Campus Amsterdam, VU University, Amsterdam, The Netherlands; 2 Molecular and Cellular Neurobiology, Centre for Neurogenomics and Cognitive Research, Neuroscience Campus Amsterdam, VU University, Amsterdam, The Netherlands; 3 Department of Clinical Genetics, VU Medical Center, Amsterdam, The Netherlands; University of Illinois at Urbana-Champaign, UNITED STATES

## Abstract

The secretory pathway in neurons requires efficient targeting of cargos and regulatory proteins to their release sites. Tomosyn contributes to synapse function by regulating synaptic vesicle (SV) and dense-core vesicle (DCV) secretion. While there is large support for the presynaptic accumulation of tomosyn in fixed preparations, alternative subcellular locations have been suggested. Here we studied the dynamic distribution of tomosyn-1 (Stxbp5) and tomosyn-2 (Stxbp5l) in mouse hippocampal neurons and observed a mixed diffuse and punctate localization pattern of both isoforms. Tomosyn-1 accumulations were present in axons and dendrites. As expected, tomosyn-1 was expressed in about 75% of the presynaptic terminals. Interestingly, also bidirectional moving tomosyn-1 and -2 puncta were observed. Despite the lack of a membrane anchor these puncta co-migrated with synapsin and neuropeptide Y, markers for respectively SVs and DCVs. Genetic blockade of two known tomosyn interactions with synaptotagmin-1 and its cognate SNAREs did not abolish its vesicular co-migration, suggesting an interplay of protein interactions mediated by the WD40 and SNARE domains. We hypothesize that the vesicle-binding properties of tomosyns may control the delivery, pan-synaptic sharing and secretion of neuronal signaling molecules, exceeding its canonical role at the plasma membrane.

## Introduction

Neural communication is established by the controlled release of signaling molecules from synaptic vesicles (SVs) and large dense-core vesicles (DCVs). Coordinated transport is essential to deliver secretory vesicles and their cargos to sites of release. For synapse formation in young neurons, multiple active zone proteins are packaged and co-transported in piccolo-bassoon transport vesicles (PTVs) [1,2], while synaptic vesicle components are transported by synaptic vesicle precursor (SVP) organelles [3,4]. Lateral axonal transport in mature neurons is central to dynamic sharing of vesicles across adjacent presynaptic boutons, implicated in synaptic plasticity [5–7]. Interestingly, vesicular organelles with different destinations co-migrate in neurites [8,9], while the final subcellular targeting steps are likely encoded by molecules on the vesicle surface [10–12].

Neurotransmitter release is mediated by a complex of VAMP2 on the vesicular membrane and syntaxin-1/SNAP25 on the plasma membrane, although the latter molecules were also observed on the vesicle surface [13–16]. Tomosyn is an inhibitor of such SNARE (Soluble NSF Attachment Protein Receptor)-mediated secretion from SVs [16–20] and DCVs [21,22] that fuse with the plasma membrane in axons and dendrites [23,24]. It competes with the vesicular SNARE for t-SNARE-binding, does not contain a vesicle-binding motif itself and was suggested to thereby prevent priming of vesicles [20,25,26]. By splice variation, two paralogous genes (tomosyn-1/STXBP5 and tomosyn-2/STXBP5L) give rise to at least seven tomosyn isoforms in the mammalian brain [27].

In line with a presynaptic function, tomosyn localizes with synaptic markers in mouse hippocampal tissue [28], hippocampal neurons in primary culture [29], superior cervical ganglion cells [17] and *C*. *elegans* motor neurons [19]. Dendritic localization has been observed in mouse hippocampal tissue slices [28]. In both HEK293 and PC12 cells, fluorescent-tagged tomosyn exhibits a diffuse cytoplasmic distribution, whereas co-expression of syntaxin-1A induces plasma membrane binding [30,31]. In insulin-secreting INS-1E cells [32] and MIN6 cells [29], tomosyn expression partly co-localizes with secretory granules. Amisyn, a tomosyn homologous protein, is mainly cytosolic, but a fraction associates with membranes in rat brain extract, partly independent of syntaxin [33]. Both tomosyn and amisyn are present on SVs according to proteomic analysis [16]. Tomosyn also associates with DCVs in *C*. *elegans* immuno-electron microscopy [21]. Thus, while there is large support for the synaptic targeting of tomosyn in fixed preparations, a number of other localizations have also been described, prompting a need for a more detailed localization of tomosyn in living neurons.

In this study we show that tomosyn is targeted to migrating SVs and DCVs by multiple redundant interactions located in different domains of the protein. These data suggest an intricate role of tomosyn beyond the conventional model in which it inhibits neurotransmitter release by competing with VAMP2 for t-SNARE binding on the plasma membrane. We hypothesize that tomosyn might function to regulate synaptic capturing of secretory vesicles and may be key when recycling vesicles are shared between presynaptic terminals.

## Materials and methods

### Neuronal cultures

Animals were housed, bred and experimentally used according to Institutional guidelines and Dutch and U.S. governmental laws with prior approval from the institutional animal research ethics committee (“Dierexperimentencommissie Vrije Universiteit/VU medisch centrum”; approval number FGA11-03), ensuring minimum discomfort of animals. All procedures were approved by the C57BL/6 wild type mice were obtained from Harlan (Horst, The Netherlands). Tom-1^KO/KO^ and Syt-1^KO/KO^ mice were described [22,34]. E18 stage embryos were obtained by Caesarean section of pregnant females from timed matings. Hippocampi were isolated in Hanks Buffered Salt Solution (HBSS; Sigma, St. Louis, MO) plus 10 mM HEPES (pH 7.5) (Invitrogen, Carlsbad, CA) at room temperature. After removal of the meninges, hippocampi were incubated in HBSS/HEPES (pH 7.5) plus 0.25% trypsin (Invitrogen) for 20 min at 37°C. The hippocampi were washed and triturated with a fire polished pipette tip to obtain a cell suspension. Cultured neurons were maintained in Neurobasal medium (Invitrogen) supplemented with 2% B-27 (Invitrogen), 1.8% HEPES (pH 7.5), 0.25% glutamax (Invitrogen) and 0.1% penicillin/streptomycin (Invitrogen). Low density cultures were generated by plating 2-10K hippocampal neurons per well (12-wells) on a coverslip with a confluent layer of glia, prepared by plating 25K/well frozen rat glia on etched glass coverslips, coated with 0.1 mg/ml poly-D-lysine (Sigma), 0.2 mg/ml rat tail collagen (BD Biosciences, Franklin Lakes, NJ) and 10.2 mM acetic acid (Sigma). For Western blot analysis, 100-150K neurons were plated per well (6-well) coated with 0.0005% poly-L-ornithin (Sigma) and 2 μg/ml laminin (Sigma, L2020). For immuno-EM, 150K neurons were plated per well (6-well) coated with 0.0005% poly-L-ornithin (Sigma) and 2 μg/ml laminin (Sigma, L2020).

### Constructs and viruses

An EYFP-tag was fused to the N-terminus of mouse tomosyn-m1 (Genbank accession number NP_001074813.2) and tomosyn-xb2 (Genbank accession number NP_766028.2). Codon optimization was used to increase tomosyn-m1 expression, with ‘gatggc’ to ‘gacggg’ transition at amino acid positions 458–459 (DG) and ‘gaactttacggc’ to ‘gagctctacgga’ at amino acid positions 671–674 (ELYG). Synapsin-mCherry was a gift from A. Jeromin (Allen Brain Institute, Seattle, USA). These constructs were cloned into a p156RRL lentiviral backbone vector and expressed by a FUW (tomosyn) or CMV (synapsin) promoter. Tomosyn WD40-tail fragment was generated from the EYFP-tomosyn-m1 encoding lentiviral vector using primers 5’-tgacaactagaactcagtaagtccagg-3’/5’-gacttactgagttctagttgtcacgggatgtgttgtgcgag-3’. The other fragments were cloned from mouse tomosyn-m1 using 5’-aagctgtacaagctcggtgaactcttcacgc-3’/5’-ttcgtctagaacttactgagttctagttgtcagaactgg-3’ (Coiled coil-tail) and 5’-aagctgtacaaaggccctggtgggatcg-3’/5’-ttcgtctagaacttactgagttctagttgtcagaactgg-3’ (Coiled coil), N-terminally fused to an EYFP-tag and subcloned into a p156RRL lentiviral backbone vector. Expression of tomosyn fragments was validated by Western blot analysis. Neuropeptide Y (NPY)-mCherry was cloned into a LentiLox 3.7 vector and expressed by a synapsin promoter. Lentiviral particles were produced as described before [35]. Viral transduction was performed at DIV1 (days *in vitro*) with >99% infection efficiency for all viruses.

### Western blot

Lysate for Western blot analysis was prepared by homogenizing cells in denaturing Laemmli sample buffer and boiling for 5 min at 100°C. Cells had been in culture for 14 days and were infected with lentivirus the day after plating. After SDS-PAGE and wet protein transfer to a PVDF membrane for 2 h at 350 mA at 4°C, nonspecific antibody binding to the membrane was prevented by incubation with blocking solution (5% w/v milk powder and 0.2% Tween-20 in TBS, pH 7.5) for 1 h at 4°C. Primary antibody incubation was done overnight at 4°C. After washing with TBS, the membrane was stained with secondary antibody conjugated with alkaline phosphatase (AP; DAKO, Glostrup, Denmark, 1:5000) for 1 h at 4°C. After washing again, the AP conjugated antibody was visualized using ECF substrate (GE Healthcare, Little Chalfont, UK). The membrane was scanned with a Fujifilm FLA-5000 Reader.

### Immunocytochemistry

Cells were fixed at DIV14 with 3.7% formaldehyde (Electron Microscopy Sciences, Hatfield, PA) and permeated with 0.1% Triton X-100 (Sigma) in PBS for 5 min at room temperature. Nonspecific antibody binding was prevented by incubation with blocking solution containing 2% normal goat serum and 0.05% Triton X-100 in PBS (pH 7.5) for 20 min. Primary antibody incubation was done for 2 h at room temperature. After washing with PBS, cells were incubated for 1 h at room temperature with Alexa dye conjugated secondary antibodies (Molecular Probes, Eugene, OR, 1:1000). Antibodies were diluted in blocking solution. After washing again, cells were mounted using Dabco-Mowiol (Calbiochem, San Diego, CA). Sequential imaging was done with a laser confocal LSM510 microscope (Zeiss, Oberkochen, Germany). Areas with minimal glial background staining were selected. Pearson’s correlation [36] and Manders’ overlap coefficients M1 and M2 [37] were calculated using ImageJ (NIH, USA) and the JACoP plugin (F.P. Cordelieres and S. Bolte).

### Immuno-electron microscopy

Hippocampal neuron cultures with or without EGFP-tomosyn-m1 overexpression were fixed (4% formaldehyde, 0.1% glutaraldehyde in phosphate buffer) at room temperature on DIV14 and prepared for cryo-sectioning according by the Tokuyasu method [38]. Briefly, the cells were scraped in gelatin and spun down to a semi-compact pellet. Specimen blocks were cut out, infused with 2.3 M sucrose at 4°C and mounted on aluminium pins by rapid freezing in liquid nitrogen. 70 nm sections were obtained at -120°C using a cryo-ultramicrotome (UC6, Leica). Sections were captured on carbon/formvar-coated copper mesh grids. Grids were immuno-labeled using anti-tomosyn-1 (#183103; SySy, Göttingen, Germany, 1:50) and protein-A-gold 10nm (PAG10; CMC Utrecht, The Netherlands) as electron-dense marker. Grids were counterstained by uranyl acetate in methylcellulose before analysis on TEM (Tecnai T12, FEI).

### Antibodies

Primary antibodies used for Western blotting were custom-made isoform-specific polyclonal anti-tomosyn-1 (#183103 directed to mouse tomosyn-1 aa. 651–741; SySy, Göttingen, Germany; 1:1000), anti-tomosyn-2 (#183203 directed to mouse tomosyn-2 aa. 828–983; SySy; 1:1000) and anti-VCP (K331; Sugita and Südhof, 2000; 1:1000). Tomosyn-1 and tomosyn-2 antibody specificity was validated using HEK293 cell lysates overexpressing tomosyn and brain lysates from Tom2^KO/KO^ mice [39] and using immunocytochemistry on wild type and Tom1^KO/KO^ cultured hippocampal neurons (S1 Fig). For immunocytochemistry, primary anti-MAP2 antibody (Abcam, Cambridge, UK; 1:1000) and SMI-312 antibody (Covance, Princeton, NJ; 1:1000) were used for detection of respectively dendrites and axons. Overexpressed fluorescently labelled tomosyn was stained with an anti-GFP antibody (AVES, #1020, 1:1000) and endogenous tomosyn-1 with the custom-made isoform-specific polyclonal by SySy (1:1000). To assess co-localization, antibodies recognizing VAMP2 (SySy, monoclonal #69.1, 1:2000), synapsin-1 (polyclonal #P610, 1:1000), syntaxin-1 (polyclonal #I379, 1:1000), munc18 (polyclonal #2701, 1:1000), synaptotagmin-1 (polyclonal #W855, 1:2000), VGLUT1 (SySy, 1:1000), bassoon (Enzo, Farmingdale, NY; 1:500), chromogranin B (SySy, 1:500) and CAPS-1 (SySy, #1013, 1:200) were used. For immunoprecipitation we used tomosyn-1 antibody from SySy as above; Syt1 antibody from DSHB (Iowa, IA, USA; mAb 30 and 48), SNAP25 antibodies from Epitomics (Burlingame, CA; Cat. 3132, 3173) and Genscript (Piscataway, NJ; Cat. A01445). Stx1a antibody was custom made by Genscript (to peptide CNPAIFASGIIMDSS).

### Live imaging

At DIV14-15, live imaging was performed on an Olympus IX81 microscope with a C9100 EM-CCD camera (Hamamatsu Photonics, Hamamatsu City, Japan) and a 40x (NA 1.3) or 60x objective (NA 1.49) in continuous perfusion of Tyrode’s medium (2 mM CaCl_2_, 2.5 mM KCl, 119 mM NaCl, 2 mM MgCl_2_, 30 mM glucose and 25 mM HEPES, pH 7.4) supplemented with 50 μM APV (Tocris, Bristol, UK) and 10 μM DNQX (Tocris) to block excitatory transmission. Images were acquired at 1 Hz with Olympus xcellence software (2010). Imaging of EYFP and mCherry signals was done sequentially. Field stimulation by parallel platinum electrodes was applied in 16 times 50 action potentials at 50 Hz with a 0.5 s interval by a Master 8 (AMPI, Jerusalem, Israel) and a stimulus generator (A385, World Precision Instruments, Sarasota, FL). Stimulation started after 30 s of prestimulation recording. Time lapse recordings showing >20 moving tomosyn puncta were included for analysis of movement directionality and cells with >50 total (both stable and moving) tomosyn puncta were included for an estimation of the amount of overlap with NPY/synapsin. Puncta movement and NPY/synapsin/tomosyn co-migration was analysed using kymographs with ImageJ (NIH, USA) and a plugin written by J. Rietdorf and A. Seitz. Puncta movement velocity was corrected for stationary periods.

### Immunoprecipitation of tomosyn-1 protein complexes

A hippocampal P2 + microsome fraction (Li et al., 2012) was mixed with an equal volume of extraction buffer (2% Triton X-100, 150 mM NaCl, 50 mM HEPES pH 7.4) and protease inhibitor (Roche Applied Science, Penzberg, Germany), and rotated at 4°C for 1 h. After centrifugation at 20,000 × g for 20 min, the pellet was re-extracted with extraction buffer (1% Triton X-100, 150 mM NaCl; 50 mM HEPES pH 7.4 and protease inhibitor). Supernatants from both extractions were pooled, centrifuged at 20,000 × g for 20 min and served as IP input. Typically 5 mg P2 + M and 10 μg antibodies were used for each IP experiment. After overnight incubation, 50 μl slurry of Protein A/G PLUS-Agarose beads (Santa Cruz Biotechnology, Dallas, TX) was added for each IP and rotated at 4°C for 1 h. The beads were spun down at 1000 × g for 1 min and washed four times in ice-cold extraction buffer (as above but with 0.1% Triton X-100). Beads with bound protein complexes were mixed with 2x SDS-PAGE loading buffer, and heated to 98°C for 5 min. Five μl 30% acrylamide was added and the mixture was incubated at RT for 30 min. Proteins were resolved on a 10% SDS polyacrylamide gel, fixed overnight and stained with Coomassie Blue for 30 min. Each sample lane was cut into five slices for in-gel digestion and peptide recovery as described [40].

### LC-MS-MS characterization of tryptic peptides

Peptides were re-dissolved in 20 μl 0.1% acetic acid and analyzed by an LTQ-Orbitrap mass spectrometer (Thermo Electron, San Jose, CA, USA) equipped with an HPLC system (Eksigent, Redwood City, CA). Samples were trapped on a 5 mm Pepmap 100 C18 (Dionex, Sunnyvale, CA) column (300 μm ID, 5 μm particle size) and then analyzed on a 200 mm Alltima C18 homemade column (100 μm ID, 3 μm particle size). Separation was achieved by using a mobile phase consisting of 5% acetonitrile, 94.9% H_2_O, 0.1% acetic acid (solvent A) and 95% acetonitrile, 4.9% H_2_O, 0.1% acetic acid (solvent B), with a linear gradient from 5 to 40% solvent B in 40 min at a flow rate of 400 nl/min. Eluted peptides were electro-sprayed into the LTQ-Orbitrap operated in a data-dependent mode. Mass spectrometric data was searched against the Uniprot proteomics database (version 2013-01-06) with MaxQuant software (version 1.3.0.5) to obtain peptides and proteins identified in each experiment, as well as their label-free abundance. Search parameters were: MS accuracy 6 ppm, MS-MS accuracy 0.5 Da, fixed modification of cysteine alkylation with acrylamide, variable modification of methionine oxidation and protein N-terminal acetylation, digestion with trypsin, protein hits containing at least one unique peptide, and false discovery rates of both peptides and proteins within 0.01.

### Statistical analysis

Statistical analysis was performed using SPSS (Version 20.0, Armonk, NY). Since data were not normality distributed (Kolmogorov-Smirnov), Mann-Whitney tests for two independent samples were applied.

## Results

### Diffuse and punctate distribution of tomosyn immunoreactivity in axons and dendrites

**Fig 1:**
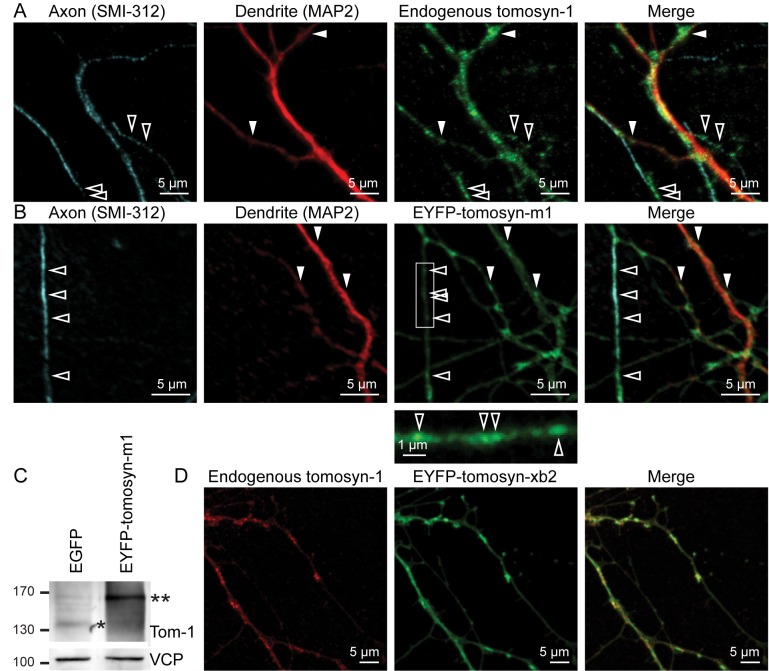
Tomosyn is distributed in a combined diffuse and punctate pattern in both axons and dendrites. Cultured hippocampal neurons were fixed at DIV14. Local accumulation of (A) endogenous tomosyn-1 (detected with a tomosyn-1 specific antibody) as well as (B) EYFP-tomosyn-m1 was observed in axons (open arrowheads) and dendrites (closed arrowheads). (C) Typical example of Western blot analysis confirming EYFP-tomosyn-m1 expression in these preparations. Asterisks indicate endogenous (*) and overexpressed (**) tomosyn-1. (D) Endogenous tomosyn-1 (red) and EYFP-tomosyn-xb2 (green) showed similar subcellular distributions.

Intracellular tomosyn-1 distribution was assessed using immunocytochemistry on primary cultures of mouse hippocampal neurons. Specificity of the custom-made antibody was confirmed by the absence of staining in Tom-1^KO/KO^ neurons (S1 Fig). As expected for a synaptic protein [17,20,28], punctate localization of tomosyn-1 was observed (Fig 1A). Interestingly, puncta were present both in axons and dendrites, supporting the previous notion that neuronal tomosyn is not confined to presynaptic sites [28]. In addition, as also reported before [20,30,31], diffuse tomosyn expression was observed.

Lentiviral expression of a N-terminal EYFP-tagged splice variant of tomosyn-1 (EYFP-tomosyn-m1; Fig 1B) yielded a similar distribution (expression levels were 3.6 ± 1.25 times higher than endogenous tomosyn-1 mean ± s.e.m.; n = 5; see typical immunoblot in Fig 1C). Expression of an EYFP-tagged splice variant of tomosyn-2 (EYFP-tomosyn-xb2) also resulted in a diffuse and punctate distribution, overlapping with endogenous tomosyn-1 (Fig 1D). Notably, EYFP-tomosyn-xb2 and endogenous tomosyn-1 did not strictly co-localize in all neurite extensions. Thus, in line with several previous observations, both tomosyn isoforms localized both in the cytosol and in clusters along neurites.

**Fig 2:**
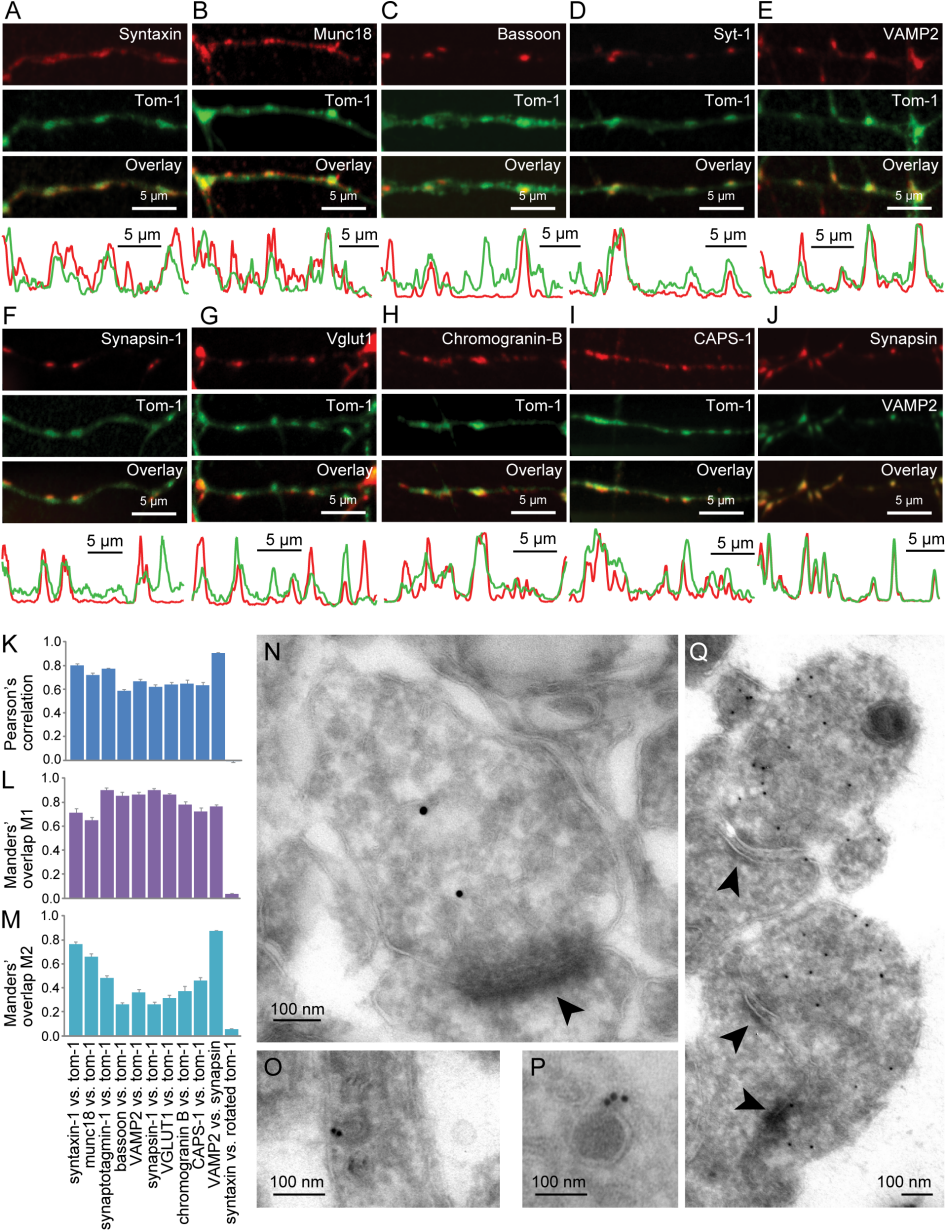
EYFP-tomosyn puncta co-localized with various synaptic and secretory vesicle markers. **(A-J)** Images show expression of EYFP-tomosyn-m1 (‘Tom-1’; green) and endogenous synaptic and secretory vesicle markers (red). Fluorescence intensity profiles along depicted neurites are displayed below each image. Co-localization of EYFP-tomosyn-m1 puncta with antibodies recognizing endogenous (A) SNARE protein syntaxin, (B) the presynaptic SNARE-associated protein munc18 as well as the active zone protein bassoon was observed, confirming presynaptic localization. Moreover, tom-1 puncta co-localized with synaptic vesicle (SV) markers (D) synaptotagmin-1 (Syt-1), (E) VAMP2 and (F) synapsin, (G) vesicular glutamate transporter-1 (VGLUT1) and (H) the DCV marker chromogranin B. (I) CAPS, implicated in release from both SVs and DCVs, additionally co-localized with tomosyn-1 puncta. (J) Synapsin / VAMP2 co-localization was used as a positive control. (K-M) Co-localization was quantified using (K) Pearson’s correlation and Manders’ overlap (L) M1 and (M) M2. As a negative control, tomosyn images were rotated relative to syntaxin. (N-Q) Ultrastructural localization of endogenous tomosyn-1 in (N) presynaptic boutons (O) vesicles in neurites and (P) dense core vesicles. (Q) Overexpressed tomosyn-1 was predominantly localized to presynaptic boutons. Arrowheads indicate post-synaptic densities.

To investigate the nature of tomosyn puncta, we performed co-localization experiments with markers for various organelles involved in synaptic function and secretory trafficking (Fig 2). Tomosyn-1 puncta co-localized with the SV proteins VAMP2 and synapsin-1 (Fig 2E and 2F), the synaptic marker bassoon (Fig 2C) and the DCV cargo protein chromogranin B (Fig 2H). However, none of these markers showed complete overlap with tomosyn-1 puncta, suggesting that tomosyn-1 expression was not restricted to any single type of organelle. The degree of co-localization between total EYFP-tomosyn-m1 (both diffuse and punctate) and the various markers was quantified by Pearson’s correlation [36] and Manders’ coefficients [37], producing the highest scores for syntaxin-1 and synaptotagmin-1 (Syt-1; Fig 2K–2M). The co-localization with VAMP2 was also observed for endogenous tomosyn-1 (S2 Fig). To achieve a higher spatial resolution, we also analyzed cultured hippocampal neurons by immune-electron microscopy. In line with the findings from light microscopy, tomosyn immunoreactivity was enriched in synaptic boutons (N-P for endogenous and 2Q for overexpressed tomosyn-1 in Fig 2) where it was either dispersed in the cytosol (Fig 2N) or associated with small clear vesicles (Fig 2O) or DCVs (Fig 2P). All in all, these results suggest that tomosyn-1 is localized not only to the cytosol, but also to (clusters of) SVs and LDCVs.

### Tomosyn-1 and -2 co-migrate with synapsin and NPY in living neurons

**Fig 3:**
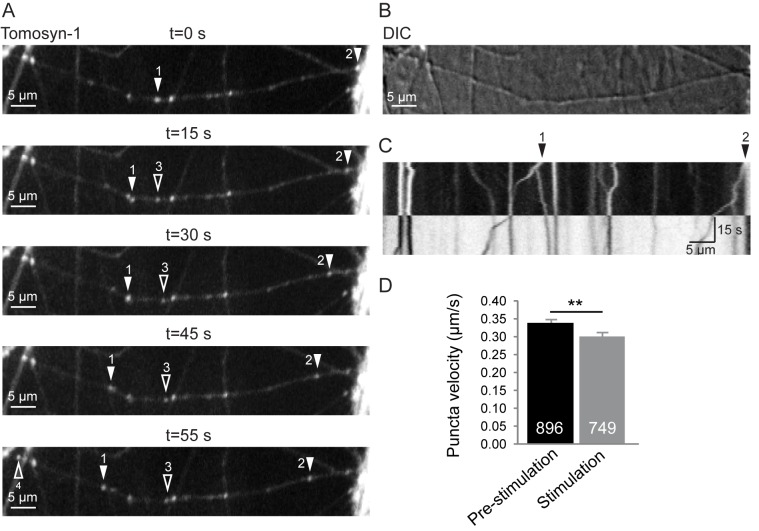
Migration of EYFP-tomosyn puncta in living neurons at DIV15. (A) Time-lapse images show bidirectional movement of EYFP-tomosyn-m1 puncta (solid arrowheads). During recording, additional puncta occasionally emerged from stable or moving tomosyn puncta (open arrowheads). After 30 s, the cells were stimulated with 16x 50 action potentials at 50 Hz. (B) DIC image of the same region. (C) Puncta movement along the neurite during the same time-lapse is depicted as a kymograph. The stimulation period is represented by inverted tones. (D) The velocity of moving tomosyn puncta reduced significantly during stimulation. Error bars depict s.e.m. and the number of analyzed vesicles is depicted in the bars. **, p<0.01.

Tomosyn localization to neuronal secretory vesicles is conceivable given its association with secretory granules in INS-1E cells [32], SVs from rat brain [16] and *C*. *elegans* DCVs [21] as well as the direct interaction of rat tomosyn with the vesicular proteins Syt-1 [41] and Rab3 [29]. To differentiate between immobile synapses and mobile organelles, we performed live imaging of EYFP-tomosyn-m1 puncta and observed that many tomosyn-1 puncta moved along the neurite (typical example in Fig 3A–3C). A kymograph representation shows bidirectional movement of these puncta (Fig 3C). In some cases, new puncta emerged from existing ones (stable or moving; open arrowheads), suggesting the segregation of vesicles from a cluster. Within 30 s, 30.3 ± 0.02% of puncta changed movement direction (mean ± s.e.m.; n = 21 cells). Since vesicular trafficking seems to be activity-dependent, puncta mobility upon neural stimulation was tested[42]. Without stimulation, the mean velocity of moving tomosyn puncta was 0.34 ± 0.01 μm/s (n = 896 puncta from 25 cells). Upon high-frequency field stimulation the speed was slightly reduced to 0.30 ± 0.01 μm/s (Fig 3C and 3D; the stimulation period is depicted by black/white inversion in Fig 3C; Mann-Whitney U = 308464.5, z = 2.823, p = 0.005, r = 0.070; n = 749 puncta from 25 cells).

**Fig 4:**
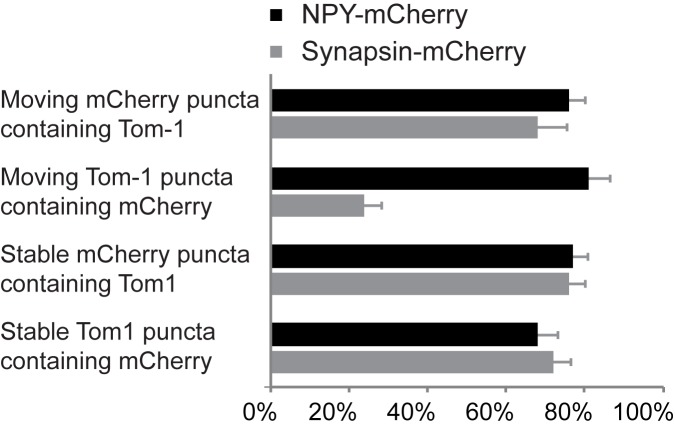
Quantification of the number of stable or mobile EYFP-tomosyn-m1 (Tom-1) puncta that contained mCherry-labelled vesicular markers and vice versa. Synapsin-mCherry (n = 10 cells) was used as a marker for SVs, whereas NPY (n = 11 cells) is a DCV cargo. Bars represent mean ± s.e.m.

To further characterize tomosyn-1 puncta, we used the genetically encoded markers synapsin-mCherry for SVs [43] and NPY-mCherry for DCVs [42] and quantified their co-localization in stable and moving puncta (Fig 4; see S1 File). In line with fixed samples (Fig 2), both markers showed a strong, but not complete co-localization with tomosyn-1 in time-lapse imaging experiments. Tomosyn-1 co-labelled 76 ± 3.9% of all stable synapsin-mCherry puncta and 68 ± 7.5% of all mobile puncta (n = 10 cells each). Using NPY-mCherry, tomosyn-1 also co-labelled 77 ± 3.6% of stable and 76 ± 4.0% of mobile puncta (n = 11 cells each). Conversely, synapsin-mCherry co-migrated with 24 ± 4.1% (n = 10 cells) of the mobile tomosyn-1 puncta, while co-labelling of NPY-mCherry was observed for 81 ± 5.2% (n = 11 cells) of the mobile tomosyn-1 puncta.

Considering that immobile synapsin-mCherry puncta likely represent this protein in synapses, these data suggest that EYFP-tomosyn-m1 accumulated at roughly 75% of the synaptic terminals, was expressed at roughly 75% of all DCVs, and co-migrated with most moving synaptic vesicles [5–7] and DCVs. Most mobile EYFP-tomosyn-m1 puncta are probably moving DCVs.

**Fig 5:**
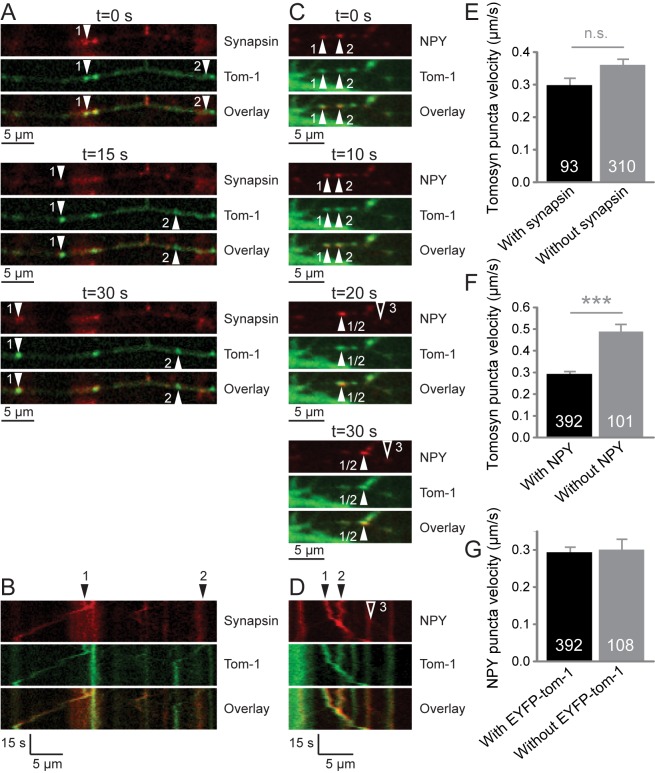
Overlap between moving EYFP-tomosyn and synapsin-mCherry/NPY-mCherry puncta in living DIV15 neurons. (A) Typical time-lapse images and (B) the corresponding kymograph show co-migration (arrowhead #1) of synapsin-mCherry (red) and EYFP-tomosyn-m1 (green) in hippocampal neurons. Also, synapsin-mCherry-negative tomosyn puncta (arrowhead #2) and EYFP-tomosyn-m1-negative synapsin puncta were observed. Similarly, (C) typical time-lapse images and (D) the corresponding kymograph are shown for NPY-mCherry co-migration. Two moving NPY-mCherry positive tomosyn puncta (arrowheads #1 and #2) and an EYFP-tomosyn-m1-negative NPY punctum (arrowhead #3) are seen in this example. Quantification of the amount of overlap is shown in Fig 4. (E) While the mean velocity of moving tomosyn puncta without synapsin-mCherry was not significantly higher than the velocity of puncta containing this SV marker, (F) puncta without NPY-mCherry moved on average faster than puncta with NPY-mCherry in the same cells. (G) Vesicular EYFP-tomosyn-1 did not affect the velocity of NPY puncta. Error bars depict s.e.m. and the number of analyzed vesicles is depicted in the bars. ***, p<0.001.

Next, we addressed the velocity of EYFP-tomosyn-m1 puncta in neurons co-expressing a mCherry-tagged marker. Typical images and kymograph representations are given in Fig 5A–5D. On the one hand, tomosyn puncta that did not co-migrate with NPY-mCherry puncta moved faster than puncta that did (Fig 5F; Mann-Whitney U = 12036, z = 6.078, p<0.001, r = 0.274; n = 392 puncta with NPY-mCherry; n = 101 puncta without NPY-mCherry; N = 13 cells). A similar trend was observed for tomosyn-1 puncta and co-migration with synapsin-mCherry (Fig 5E; Mann-Whitney U = 13261.5, z = 1.171, p = 0.242, r = 0.058; n = 93 puncta with synapsin-mCherry; n = 310 puncta without synapsin-mCherry; N = 12 cells). Possibly, the fast-moving tomosyn-1 puncta represent a third type of structure. On the other hand, co-localization/co-migration of EYFP-tomosyn-m1 did not affect the average velocity of NPY-mCherry puncta (Fig 5G). Thus, overexpressed tomosyn is unlikely to regulate the trafficking speed of secretory vesicles.

**Fig 6:**
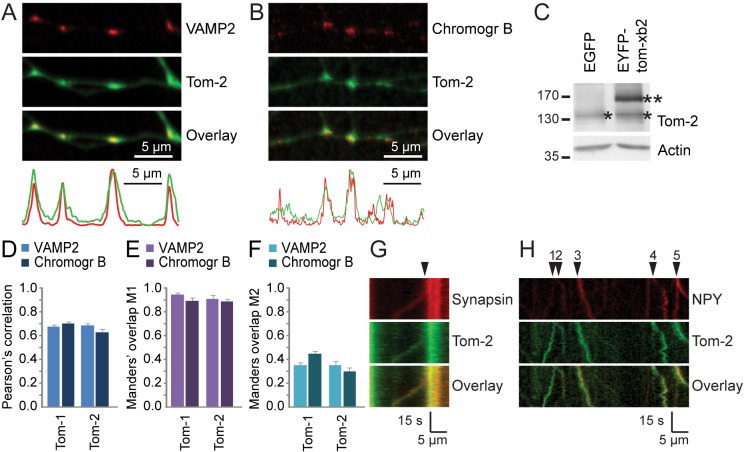
Vesicular localization of tomosyn-2. Co-localization of EYFP-tomosyn-xb-2 (‘Tom-2’; green) puncta with antibodies recognizing endogenous (A) VAMP2 and (B) chromogranin B (red) was observed. Fluorescence intensity profiles along the depicted neurites are given below the images. (C) Expression of EYFP-tomosyn-xb2 in these preparations was verified by Western blotting. Asterisks indicate endogenous (*) and overexpressed (**) tomosyn-2. Co-localization was quantified using (D) Pearson’s correlation and (E) Manders’ overlap M1 and (F) M2. Co-migration of EYFP-tomosyn-xb2 puncta (arrowheads) with (G) synapsin-mCherry and (H) NPY-mCherry was observed in living neurons, as seen in these kymograph examples.

In line with similar expression patterns for tomosyn-1 and tomosyn-2 (Fig 1D), overlap with the vesicular markers VAMP2 and chromogranin B was observed for EYFP-tomosyn-xb2 puncta (further designated as ‘tomosyn-2’; Fig 6A and 6B). Expression of the tomosyn-2 construct was validated by Western blotting (Fig 6C; EYFP-tomosyn-xb2 levels were 1.4 ± 0.18 [mean ± s.e.m.; n = 2] times the level of endogenous tomosyn-2). Quantification by Pearson’s correlation and Manders’ coefficients further indicated that the co-localization with vesicular markers was similar for tomosyn-1 and tomosyn-2 (Fig 6D–6F). Tomosyn-2 puncta co-migrating with the SV marker synapsin-mCherry (Fig 6G) and the DCV marker NPY-mCherry (Fig 6H) were both detected. We conclude that vesicular targeting is a conserved property of both isoforms.

### Molecular mechanism of vesicular tomosyn-1 targeting

The vesicular accumulation of tomosyn could involve various molecular interactions. The proteinaceous surface of synaptic vesicles purified from rat brain has been thoroughly characterized [16] and contains four known tomosyn-1 interactors: SNAP25, syntaxin-1, Syt-1 [25,41,44,45] and Rab3 [29]. The interaction with SNAP25 and syntaxin-1 involves the C-terminal coiled-coil (CC) domain of tomosyn which can engage in a stable four-helical bundle [25]. The other two interactions are both mapped to the large N-terminal domain [41,46]. While the known protein interactions offer plausible possibilities for vesicle binding, we explored the synaptic interactome for potential novel interactions and performed a series of immunoprecipitation (IP) experiments from mouse brain synaptosomes, followed by mass spectrometry (MS) to identify each interactor. To consider the most robust interactions, we focused on interactions that were confirmed in a reciprocal experiment (i.e. with swapping the bait and prey proteins). IP-MS supported the previously established tomosyn-1 interaction with syntaxin-1a (Stx1A), SNAP25 and Syt-1 in multiple independent experiments [25,41,44,45]. Reverse IP-MS analysis of these proteins confirmed the presence of tomosyn-1 (see Table 1, summarizing the intensity-based quantification or iBAQ values of interactors identified by mass spectrometry). Despite clear evidence from previous studies, our approach did not detect the Rab3 interaction, possibly because this interaction is dependent on GTP activation. Furthermore, this approach did not identify novel interactions.

**Table 1:** Summary of tomosyn-interactors identified in a series of co-immunoprecipitation experiments from mouse synaptosomes.

IP experiment^a^			Intensity-based quantification (iBAQ) values for prey proteins^b^					
Bait protein	Antibody		Tom-1	Tom-2	SNAP25	Stx1a	Stx1b	Syt1
**Tom-1**	SySy	#183103	3,363,700	252,25	246,43	363,85	2,734,900	44,672
**SNAP25**	GenScript	#A01445	1,147,100	1,942,300	319,504,000	14,469,000	31,596,000	11,864,000
**SNAP25**	GenScript	#A01445	702,81	1,441,900	157,491,100	12,627,000	24,664,000	10,099,000
**SNAP25**	GenScript	#A01445	516,88	1,434,500	131,435,000	7,107,400	16,512,000	5,694,200
**SNAP25**	Epitomics	#3132	512,09	426,201	83,411,000	6,212,900	15,857,000	3,163,200
**SNAP25**	Epitomics	#3132	2,070,700	1,980,371	129,684,900	12,722,000	43,415,000	5,318,600
**SNAP25**	Epitomics	#3173	898,69	999,813	155,734,100	7,519,800	20,364,000	3,585,800
**Stx1a**	Genscript	custom	552,62	134,39	709,835	6,482,800	7,235,400	658,58
**Stx1a**	Genscript	custom	45,465	28,163	1,126,192	3,438,800	639,61	334,93
**Stx1a**	Genscript	custom	52,996	29,837	1,756,115	3,629,400	622,47	183,2
**Stx1a**	Genscript	custom	35,387	44,449	993,02	3,759,800	707,8	86,825
**Syt1**	DSHB	mAb 30	67,214	4,542	1,842,200	885,44	1,943,100	153,500,000
**Syt1**	DSHB	mAb 30	12,309	0	606,11	143,25	737,24	72,604,000
**Syt1**	DSHB	mAb 30	5,503	3,049	844,43	255,42	982,94	109,688,834
**Syt1**	DSHB	mAb 48	67,531	5,719	813,46	1,101,400	1,711,400	144,450,000
**Syt1**	DSHB	mAb 48	13,783	1,878	901,28	231	1,040,800	99,027,000
**Syt1**	DSHB	mAb 48	7,269	2,96	736,96	410,67	1,505,400	114,177,859

^a^Each row represents a single IP experiment with antibodies directed to the indicated bait protein.

^b^The iBAQ values are summarized for all splice isoforms from each gene (Stxbp5: Q8K400,D3Z079,D3Z2Q2 and F6WXQ4; Stxbp5l: Q5DQR4;Q5DQR4-2;Q5DQR4-3;Q5DQR4-4 and Q5DQR4-5; SNAP25: Uniprot P60879 and P60879-2; Stx1a: O35526 and D6RFB9; Stx1b: P61264; Syt1: P46096 and D3Z7R4).

**Fig 7:**
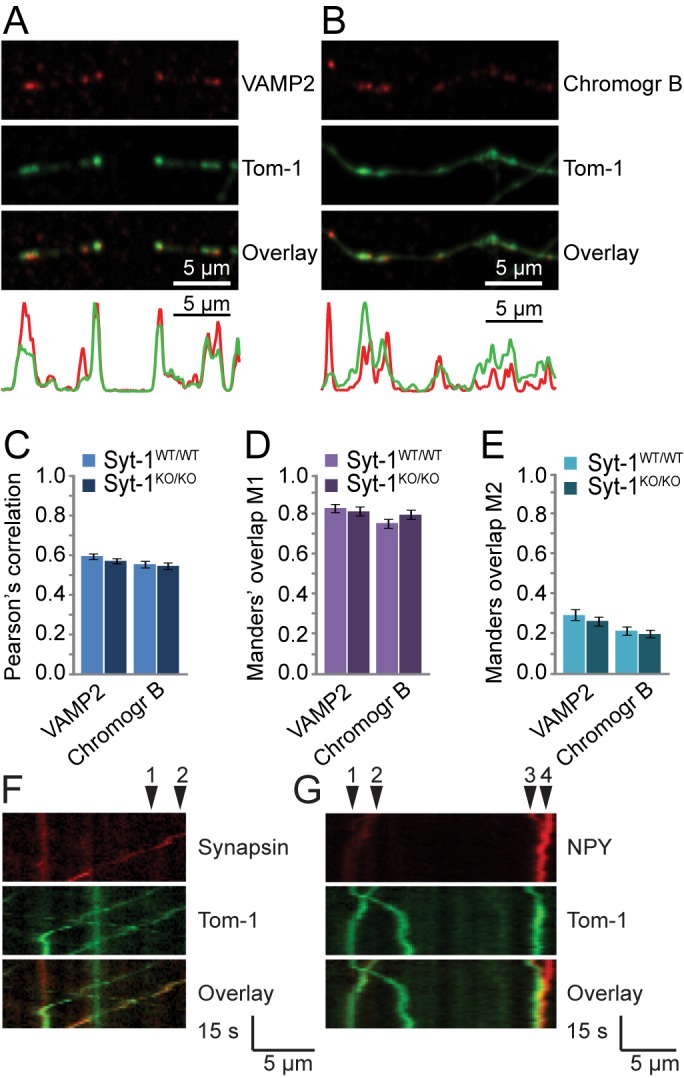
NPY and synapsin co-migration with tomosyn-1 was unaffected in Syt-1^KO/KO^ neurons. (A-B) EYFP-tomosyn-m1 (‘Tom-1’; green) puncta co-localized with (A) synaptic vesicle marker VAMP2 and (B) DCV marker chromogranin B (both depicted in red) in DIV14 Syt-1^KO/KO^ neurons. Fluorescence intensity profiles along the neurites are shown below the images. (C-E) Overall co-localization of VAMP2 vs. Tom-1 (‘VAMP2’) or of chromogranin B vs. Tom-1 (‘Chromogr B’) was quantified using (C) Pearson’s correlation, (D) Manders’ overlap M1 and (E) M2, which were similar in wild type and Syt-1^KO/KO^ neurons. Furthermore, mobile EYFP-tomosyn-m1 puncta (green) co-migrated with (F, arrowheads) synapsin-mCherry (‘Synapsin’; red) and (G, arrowheads #1–3) NPY-mCherry (‘NPY’; red) in Syt-1^KO/KO^ neurons.

In view of the strong co-localization with Syt-1 (Fig 2D) and its important role in secretion via both SVs [34,47] and DCVs [48,49], we first tested whether the vesicular co-localization of tomosyn depends on the presence of Syt-1. Co-localization and co-migration of EYFP-tomosyn-m1 with vesicular markers was assessed in Syt-1 deficient (Syt-1^KO/KO^) hippocampal neurons. These neurons completely rely on Syt-1 for synchronous synaptic transmission [34]. The amount of co-localization between tomosyn-1 and VAMP2 or chromogranin B was unaffected (Fig 7A–7E). Moreover, co-migration of tomosyn-1 puncta with synapsin-mCherry (Fig 7F) and NPY-mCherry (Fig 7G) was still observed in absence of synaptotagmin-1: in Syt-1^WT/WT^ neurons, 78 ± 4.1% moving NPY puncta contained tomosyn (n = 9 cells), compared to 76 ± 4.2% in Syt-1^KO/KO^ neurons (n = 8 cells). Thus, even though Syt-1 is a known tomosyn-1 interactor, it is not essential for its vesicular targeting.

**Fig 8:**
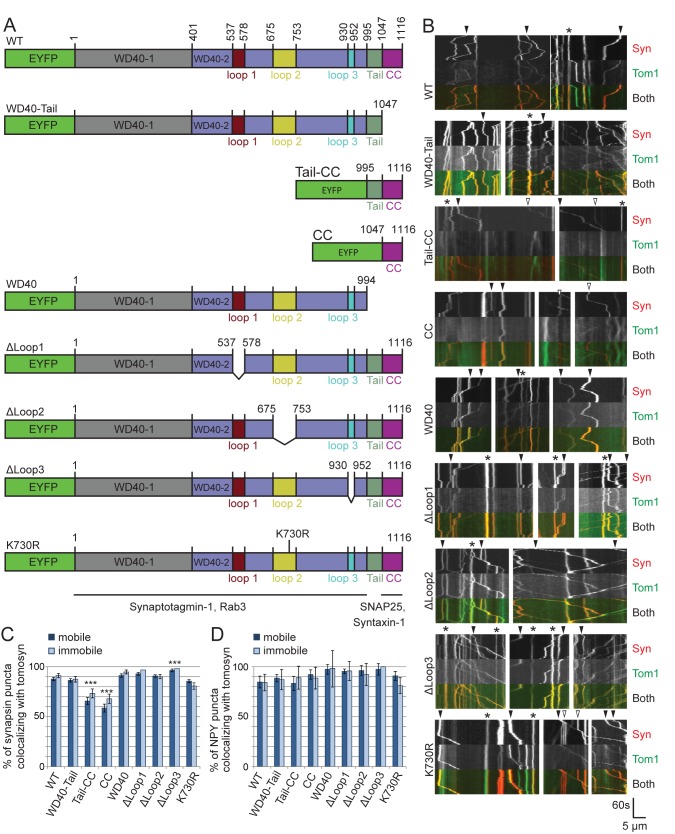
The vesicular co-localization of tomosyn involves redundant interactions in the N- and C-terminal domains. A) Wild type and mutant EYFP-tomosyn-1m constructs were co-expressed with synapsin-mCherry or NPY-mCherry using lentiviral vectors. mCherry-labelled puncta were observed by live imaging during 60s at 1 frame/s. Previously mapped interaction domains with Syt-1, Rab3, SNAP25 and syntaxin-1 are indicated by solid black lines below the constructs [25,29,41,44–46]. B) Representative examples of EYFP-tomosyn-1m (Tom1) and synapsin-mCherry (Syn) dynamics in neurites depicted as kymographs for each construct. In all groups, co-migration of EYFP and mCherry was observed in mobile puncta (some examples are indicated by closed arrowheads). Open arrowheads indicate mobile mCherry puncta with no detectable EYFP-tomosyn fluorescence. Asterisks indicate immobile double-labelled structures. C) Quantitation of the percentage of synapsin-mCherry puncta that showed detectable EYFP-tomosyn fluorescence. Both mobile and immobile structures are displayed. Data are presented as mean ± s.e.m from n = 37–45 cells and 1940–3090 puncta. Statistical tests were performed for mobile synapsin-containing puncta. The strongest reduction in the percentage of tomosyn-labelled puncta was observed after deletion of the N-terminal domain (see constructs “Tail-CC” and “CC”, ***; p<0.001). D) Similar quantitation data for NPY-mCherry puncta calculated from n = 17–21 cells and 373–912 puncta).

Likewise, we probed whether tomosyn-1 interaction with the SNAREs syntaxin-1 and SNAP25 is essential for its vesicular targeting. This interaction depends on tomosyn’s C-terminal SNARE domain [44]. Lentiviral expression vectors for various tomosyn fragments carrying C-terminal deletions (Fig 8A) co-migrated normally with SVs and DCVs as shown by live imaging (see constructs “WD40-Tail” and “WD40” in Fig 8; see S2 and S3 Files), suggesting that SNARE interactions are not essential for vesicular targeting of tomosyn.

To further investigate the potential role of interactions involving the tomosyn WD40-1 and WD40-2 domains (Rab3 and Syt-1), we also investigated the effect of N-terminal or internal deletions on tomosyn’s vesicular co-localization. All tested EYFP-tomosyn constructs were observed to co-migrate with synapsin-mCherry and NPY-mCherry (Fig 8B) to some extent. Quantitative analysis showed a reduced percentage of EYFP-positive synapsin-mCherry puncta in cells that expressed the tomosyn fragments named “Tail-CC” and “CC” (Fig 8C; Mann-Whitney U = 350; z = -4.662 for WT vs Tail-CC and U = 245; z = -5.239 for WT vs CC; n = 42 cells for WT, 41 for Tail-CC and 37 for CC), suggesting that tomosyn’s N-terminal domain contributes to synaptic vesicle binding. Nevertheless, the SNARE domain fragment (residues 1047–1116 of tomosyn; CC) still co-localized with more than 50% of the synapsin-mCherry puncta (see arrowheads in Fig 8B; see also S4 and S5 Files). According to structural models, three loops are predicted to emanate out of the N-terminal domain structure [31]. Deletion of each of these loops separately (Δloop1, Δloop2, Δloop3), as well as mutation of the SUMOylation site located in loop 2 at residue K730 (K730R), did not block vesicle targeting. Similar results were obtained for large dense-core vesicles labelled by NPY-mCherry, except that the constructs Tail-CC and CC did not show a reduced percentage of tomosyn-positive mCherry puncta (Fig 8D).

**Fig 9:**
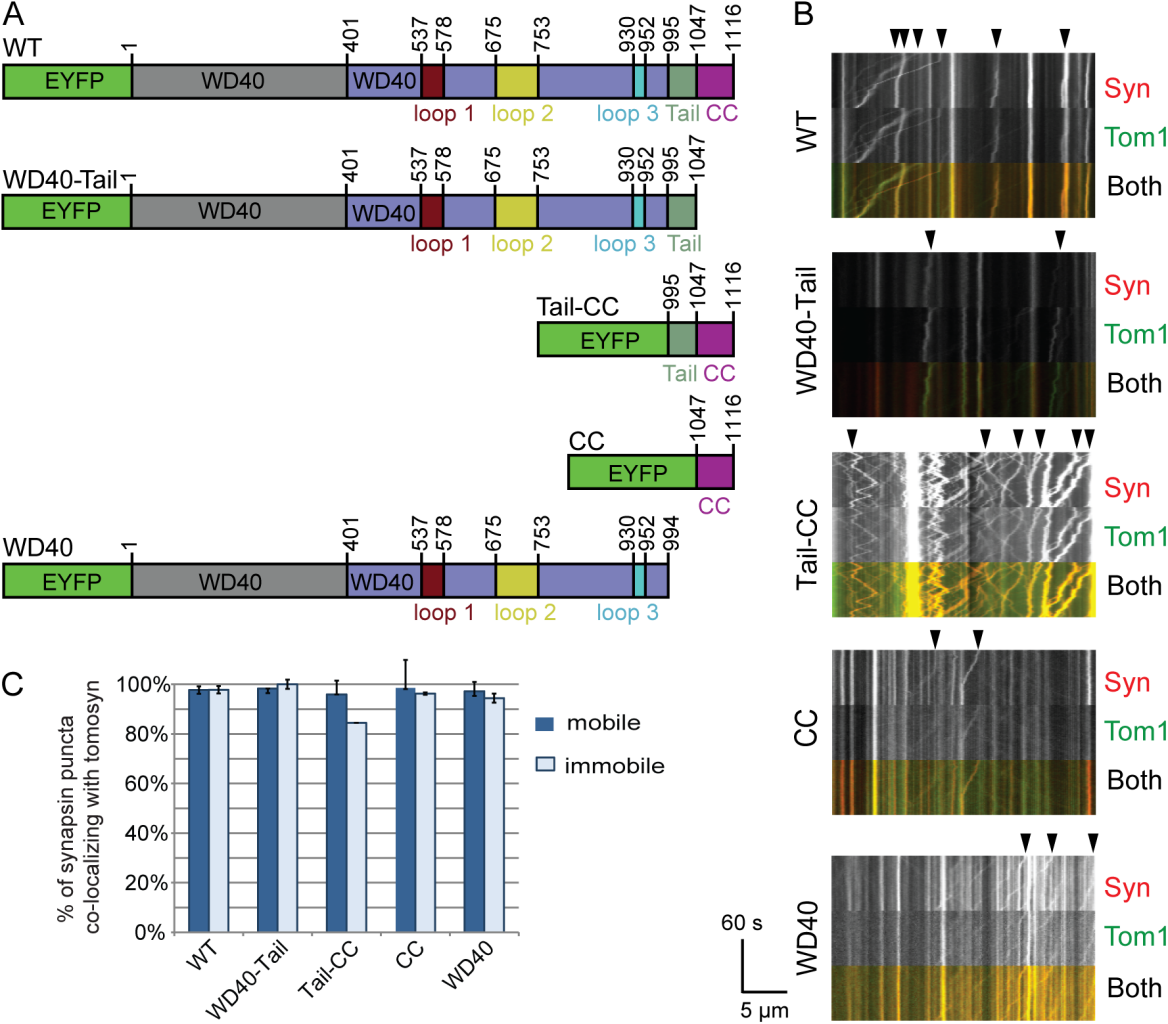
Co-migration of full-length or truncated EYFP-tomosyn constructs in Syt-1^KO/KO^ neurons. A) Wild type and mutant EYFP-tomosyn-1m constructs were co-expressed with synapsin-mCherry using lentiviral vectors. B) Representative examples of EYFP-tomosyn-1m (Tom1) and synapsin-mCherry (Syn) dynamics, depicted as kymographs for each construct. None of the constructs analyzed showed a loss of vesicular co-migration. C) Quantitation of the percentage of immobile and mobile synapsin-mCherry puncta that co-labeled EYFP-tomosyn fluorescence. Statistical tests were performed for mobile synapsin-containing puncta. Data show mean ± s.e.m from N = 9 cells and 290–396 puncta.

In a next experiment, we tested whether the vesicular accumulation could be the result of two redundant interactions: one via Syt-1 and another via a SNARE-pairing mechanism on tomosyn’s C-terminal domain. Even in Syt-1^KO/KO^ neurons, the lack of the C-terminal Tail and CC domains did not abolish the co-migration of tomosyn fragments with synapsin-mCherry (Fig 9B, see arrowheads). Quantitative analysis showed that the degree of co-localization with synapsin-mCherry was still higher than 80% in all tested constructs (Fig 9C, data from 9 cells with 369 puncta for the WT construct, 303 for WD40-Tail, 364 for Tail-CC, 241 for CC and 290 for WD40). In this smaller experiment, the Tail-CC construct showed a similar trend towards a lower degree of co-localization as in Syt-1 wildtype cells (compare with Fig 8C), where the “CC” construct showed near-complete SV association. Taken together, these data show that tomosyn binds to secretory vesicles by a mechanism that does not require Syt-1.

## Discussion

Tomosyn is a cytosolic inhibitor of secretion that localizes both pre- and postsynaptically in diverse model systems [17,20,28]. Despite the absence of a membrane anchor, some evidence points to an association of tomosyn with secretory vesicles [16,21,32]. Here we studied the localization of tomosyn in cultured hippocampal neurons. Besides a diffuse distribution in neurites and accumulation at synapses, tomosyn co-localized with moving SVs and DCVs in living neurons. The presence of at least a third type of tomosyn-containing transport organelles was suggested by fast-moving tomosyn puncta that did not co-migrate with synapsin- or NPY-mCherry (Fig 5E and 5F). In line with the broad distribution of neuronal secretory vesicles [42,50], tomosyn puncta were observed in both axons and dendrites. The association of tomosyn with secretory vesicles is apparently driven by multiple redundant interactions in the N- and C-terminal domains. The observation that the SNARE domain alone is sufficient for co-migration with secretory vesicles suggests a contribution of the reported SNARE interaction [25,44,45,51]. In addition however, the isolated N-terminal domain is also able to bind to vesicles. This activity is not attributable to Syt-1 binding [41], leaving Rab3 as the most likely candidate [29]. Interestingly, the yeast tomosyn ortholog Sro7p also associates with secretory vesicles through an interaction with the Rab GTPase Sec4p [46,52]. This interaction is GTP-dependent and was mapped to the boundary between the two WD40 propellers of Sro7p, suggesting a highly conserved role in vesicular trafficking. Compared to full length tomosyn, reduced co-migration of tail-CC and CC tomosyn-m1 fragments with synapsin puncta, but not NPY puncta, was observed, suggesting that the tomosyn binding modes may differ between SVs and DCVs.

### Experimental design influences the measured velocity of neuronal vesicle transport

Velocities of axonal Sema3A containing DCVs (~0.8 μm/s) [42], axonal unidirectional neuropeptide Y (NPY) puncta (~0.75–1.14 μm/s) [53] and axonal synaptophysin-containing vesicles (~0.69 μm/s) [54] are higher than the velocities of moving puncta in the current study (synapsin: 0.30 ± 0.022 μm/s [n = 93 puncta from 12 cells]; NPY: 0.29 ± 0.012 μm/s [n = 500 puncta from 13 cells]). During stimulation however, NPY puncta velocity (0.27 ± 0.013 μm/s [n = 401 puncta from 13 cells]) was comparable to Sema3A containing vesicles [55]. Notably, vesicular movement kinetics differs between axons and dendrites [50,55]. Although movement of individual puncta was clearest in thinner neurites, likely to be axons, our experimental conditions did not allow to unequivocally identify these compartments. Of additional importance, our experiments were performed at room temperature whereas others have determined vesicle mobility using live imaging setups equipped with a heating chamber [42,53]. Thus, different experimental designs are likely to underlie the variation in reported speed of vesicular transport.

### Functional implications

The ability to associate with the vesicle membrane should be taken into account in functional models for tomosyn-dependent secretory regulation. As suggested by previous findings, tomosyn could contribute to organelle trafficking in several ways: (i) by regulating vesicle motility through interactions with motor proteins, or (ii) by directing SNARE-dependent vesicle tethering/docking to their correct target sites.

The first hypothesis is supported by studies in yeast, where the tomosyn orthologue Sro7p [56] interacts with the actin-binding motor protein myosin Va, implicated in polarized exocytosis [57]. In neurons, Myosin Va regulates retrograde axonal transport of DCVs [53] as well as local movement of SVs [58]. In our experiment using synaptosomes however, type V myosin did not co-immunoprecipitate with tomosyn-1 or -2. Anterograde transport of SV proteins is mediated by the neuron-specific kinesin motor protein KIF1A [59,60]. In *C*. *elegans* ventral cord motor neurons, axonal targeting of tomosyn is regulated by the KIF1A homolog Unc-104 [20,61]. Again in our synaptosome preparation, KIF1A did not co-IP with tomosyn-1 or -2. We also did not detect an effect of overexpressed tomosyn on the velocity of vesicles (Fig 5G). Thus, whereas the essential role of motor proteins in vesicle trafficking is undisputed, our collective data suggests no major role for tomosyn in regulating the activity of motor proteins in mammalian hippocampal neurons.

According to the second hypothesis, tomosyn may co-migrate with secretory organelles to inhibit release at off-target sites and support their delivery at the correct destination. In mature synapses, recycling SVs are shared between release sites [5–7], a process that could contribute to synaptic plasticity during repetitive stimulation. In our study we frequently observed the stopping of moving tomosyn puncta at an immobile fluorescent structure. This, as well as the observed co-localization with bassoon, suggests that the immobile structures are likely synapses. We also observed events where a single moving fluorescent structure split two or more fluorescent structures, or vice versa (see a few examples in Fig 8). This supports the existing idea that vesicles can be co-transported in clusters [7]. In a recent study in insulin-secreting INS-1 cells, tomosyn tightly associated with NPY-GFP labeled DCVs where it remained associated until near the time of vesicle fusion and then diffused away [62]. Taken together, these observations support the hypothesis that tomosyn contributes to the trafficking and delivery of secretory vesicles.

### Potential mechanisms for tomosyn-mediated cargo delivery

Which mechanism could be responsible for capturing secretory vesicles at their correct destinations? First, the interaction of VAMP2, the vesicular SNARE, is thought to contribute to this specificity by forming trans-complexes with its cognate t-SNAREs, syntaxin-1 and SNAP25 [63]. However, trans-SNARE complex formation may be preceded by molecular interactions of Syt-1 with the t-SNARE complex [64,65]. If tomosyn associates with both Syt-1, syntaxin-1 and SNAP25 during their vesicular transport, it is conceivable that the higher concentrations of t-SNAREs at release sites [13–16] may induce tomosyn-mediated capturing of secretory vesicles by forming a tethering complex between Syt-1, tomosyn itself and the t-SNARE complex. Such complexes have indeed been detected in the LP2 fraction from rat cerebral cytosol [66] and in direct pulldown experiments [41]. Subsequently, tomosyn may aid transitioning to an exocytic SNARE complex [67].

If tomosyn interacts with proteins on the destination site to deliver vesicles at their release sites, this would presumably involve significant conformational changes. Structural and functional studies of tomosyn and its yeast orthologue Sro7p have suggested a model where the N-terminal WD40 domains can support intramolecular interactions with the C-terminal domain [31,52,56]. Intramolecular rearrangements likely affect the accessibility of the SNARE domain for t-SNARE pairing and for tomosyn’s activity in regulating neurotransmission [68]. Posttranslational changes in tomosyn or its binding partners, such as the phosphorylation of tomosyn by PKA [17], by Cdk5 via interaction with the GTP-bound state of Rab3A [29] or of syntaxin-1 by the Rho/ROCK pathway [69] could affect the likelihood of such structural rearrangements.

In conclusion, tomosyn is historically thought to be a soluble inhibitor of vesicular transmitter release that hampers vesicle fusion by v-SNARE competition. In this study, tomosyn localization to secretory and/or transport vesicles was observed, which challenges this classical view. Besides co-transport with other proteins engaged in secretion, vesicular tomosyn might be involved in spatial restriction of vesicle fusion and synaptic capturing of secretory vesicles.

## Supporting information

S1 Fig Anti-tomosyn-1 antibody was specific to tomosyn-1. (A) In wildtype DIV14 hippocampal neurons, tomosyn-1 immunoreactivity showed a distribution similar to VAMP2. (B) As a control, tomosyn-1 showed limited immunoreactivity in Tom-1^KO/KO^ neurons.
(EPS)

S2 Fig Both endogenous and overexpressed tomosyn-1 puncta co-localize with VAMP2 in cultured hippocampal neurons. (A) Immunoreactivity of endogenous tomosyn-1 (green) and VAMP2 (red) and quantitation of both staining intensities along a neurite (bottom). (B) Similar comparison of EYFP-tomosyn-m1 fluorescence (green) and endogenous VAMP2 immunoreactivity (red).
(EPS)

S1 File Live imaging movie of EYFP-tomosyn-m1 (green) and synapsin-mCherry (red) in DIV15 neurons. Dual-colour images were acquired for 60 frames at 1 Hz.
(MP4)

S2 File Live imaging movie of construct “WD40-Tail”. EYFP-tomosyn-1 and synapsin-mCherry are depicted in green and red, respectively. Dual-colour images were acquired for 60 frames at 1 Hz.
(MP4)

S3 File Live imaging movie of construct “WD40”. EYFP-tomosyn-1 and synapsin-mCherry are depicted in green and red, respectively. Dual-colour images were acquired for 60 frames at 1 Hz.
(MP4)

S4 File Live imaging movie of construct “Tail-CC”. EYFP-tomosyn-1 and synapsin-mCherry are depicted in green and red, respectively. Dual-colour images were acquired for 60 frames at 1 Hz.
(MP4)

S5 File Live imaging movie of construct “CC”. EYFP-tomosyn-1 and synapsin-mCherry are depicted in green and red, respectively. Dual-colour images were acquired for 60 frames at 1 Hz.
(MP4)
